# Evaluating the Effect of a Sleep Prehabilitation Intervention in Patients Awaiting Elective Surgery: Protocol for a Single‐Blind Randomised Trial

**DOI:** 10.1111/jsr.70173

**Published:** 2025-08-30

**Authors:** Daniel Sibley, Ian Randall, S. Nicole Culos‐Reed, P. Maxwell Slepian, Mandeep Singh, Daniel Santa Mina

**Affiliations:** ^1^ Faculty of Kinesiology and Physical Education University of Toronto Toronto Ontario Canada; ^2^ Department of Anesthesia and Pain Management University Health Network Toronto Ontario Canada; ^3^ Faculty of Medicine University of Toronto Toronto Ontario Canada; ^4^ Faculty of Kinesiology University of Calgary Calgary Alberta Canada; ^5^ Department of Oncology, Cumming School of Medicine University of Calgary Calgary Alberta Canada; ^6^ Department of Psychosocial Resources, Arthur Child Comprehensive Cancer Centre, Cancer Care Alberta Health Services Calgary Alberta Canada

**Keywords:** exercise physiology, nutrition, prehabilitation, protocol, sleep

## Abstract

Adequate sleep health is critical for surgical recovery. Disrupted sleep can impede wound healing and cognitive performance and contribute to poor surgical outcomes. Preoperative intervention aimed at improving surgical outcomes is often referred to as prehabilitation and commonly uses exercise, nutrition or psychological intervention. Sleep prehabilitation interventions have not yet been studied. This randomised assessor‐blinded trial will measure the effect of a personalised sleep prehabilitation (PSP) intervention in addition to standard of care prehabilitation (PREHAB) on participant sleep health compared to PREHAB alone (Clinicaltrials.gov ID: NCT06762639). One hundred fifty‐four English‐speaking patients from the University Health Network's Prehabilitation Program with sleep disturbance and a surgery within 4–12 weeks will be recruited. Patients will be excluded if they are participating in PREHAB remotely, have an existing sleep disorder, are a shift worker, have travel plans outside of their usual time zone or have a cognitive disability that precludes participation. Study assessments occur at baseline, 1 week before surgery and 6 weeks after surgery. PREHAB consists of individualised exercise and nutrition support as well as psychological intervention. The PSP consists of a baseline sleep assessment, brief behavioural treatment for insomnia (BBTI), sleep hygiene and behaviour‐change support. The primary outcome is the Pittsburgh Sleep Quality Index (PSQI). The primary analysis will be an ANCOVA to detect differences in PSQI between groups 1 week before surgery whilst controlling for baseline scores. The proposed study will be the first to explore the effect of a personalised preoperative sleep intervention.

## 1 Introduction

Sleep health is a well‐established determinant of overall health. Short sleepers (< 7 h per night) experience higher rates of diabetes, hypertension, cardiovascular disease, obesity and mortality (Itani et al. 2017). In the perioperative period, adequate sleep is requisite for optimal immune function, wound healing, energy metabolism and cognitive performance (Mukherjee et al. 2015). Impairment of these processes due to inadequate sleep can impede recovery and contribute to poor surgical outcomes (Gillis et al. 2022). For example, a recent meta‐analysis of 18 studies demonstrated that pre‐ or postoperative sleep disturbance substantially increased the risk for postoperative delirium (OR: 3.7; 95% CI: 2.3–6.0) (He et al. 2022). Disrupted preoperative sleep is also an independent risk factor for morbidity and a longer hospital length of stay (Kehlet 1997). An observational study of total hip arthroplasty patients showed that preoperative sleep quality predicted opioid consumption during the first 24 h after surgery (β = 0.009 [95% CI 0.002–0.015] mg/kg, *p* = 0.007, R^2^ = 0.15) and subjective pain assessment 6 months after surgery (β = 0.091 [95% CI 0.001–0.181], *p* = 0.048, R^2^ = 0.35) (Bjurström et al. 2021). Patients with obstructive sleep apnea, a condition resulting in impaired sleep quality, experience a 47% increased risk for major cardiac and pulmonary complications (Memtsoudis et al. 2014). Despite the relationship between preoperative sleep health and postoperative outcomes, sleep health disturbances are not routinely measured or addressed (Wolfe et al. 2016). This is alarming given that 60% of patients have disturbed preoperative sleep (Butris et al. 2023).

Increased pain, morbidity and hospital length of stay due to disturbed sleep health may result in increased patient and healthcare resource burden. Accordingly, optimising preoperative sleep health is a promising avenue for improving surgical care. Different approaches have been used to improve sleep health before or after surgery, though the majority of interventions have been delivered in the acute postoperative period (Machado et al. 2017). One systematic review of 10 randomised controlled trials (RCTs) used non‐pharmacological interventions such as relaxation techniques (e.g., massage), equipment or devices (e.g., music) and education to improve sleep in the acute postoperative period amongst cardiac patients (Machado et al. 2017). Whereas these data highlight the ability of interventions to mitigate or recover losses to sleep health, the preoperative period may represent a time to improve sleep health such that there is resilience to the deficit to sleep health incurred with surgery. Further, several documented causes of poor sleep postoperatively may be most appropriately addressed preoperatively, including pain, anxiety and inactivity (Memtsoudis et al. 2018). As such, intervening on sleep health exclusively in the postoperative period may miss a salient opportunity to improve surgical care.

Preoperative intervention aimed at improving surgical outcomes is often referred to as prehabilitation. Prehabilitation may include one or a combination of preoperative exercise, nutrition and psychological intervention to improve physical, psychosocial and clinical outcomes before and after surgery. While these modalities have been associated with improved sleep health outside of the surgical context, a recent RCT of prehabilitation versus usual care revealed no differences in sleep health (Atoui et al. 2023). As such, targeted sleep interventions initiated preoperatively are warranted.

Cognitive behavioural therapy for insomnia (CBT‐I) is the gold‐standard non‐pharmacological intervention for sleep disturbance (Brasure et al. 2016). Meta‐analyses have established and confirmed CBT‐I as an effective treatment for insomnia (van der Zweerde et al. 2019; Hofmann et al. 2012). Brief behavioural treatment for insomnia (BBTI) is a derivative of CBT‐I that may be particularly useful within prehabilitation due to its expeditious delivery without the requirement for specialised interventionists. A recent systematic review of 47 preoperative interventions that measure a sleep outcome revealed no interventions employed CBT‐I or BBTI (Sibley et al. 2024). As such, despite the high prevalence of preoperative sleep disturbance and the availability of established, effective non‐pharmacological treatments, no study has assessed the independent effect of an individualised sleep intervention before surgery.

## 2 Objectives

The primary objective of this study is to evaluate the effectiveness of a personalised sleep prehabilitation (PSP) intervention in addition to standard‐of‐care multimodal prehabilitation (PREHAB) on participant sleep health versus standard‐of‐care multimodal prehabilitation alone (i.e., PSP+PREHAB vs. PREHAB). Secondary objectives include examining the impact of the intervention on anthropometric, physical fitness, patient‐reported, clinical and other sleep health outcomes. A full list of study research questions is available in Appendix S1.

## 3 Methods

### 3.1 Study Design and Setting

**FIGURE 1:**
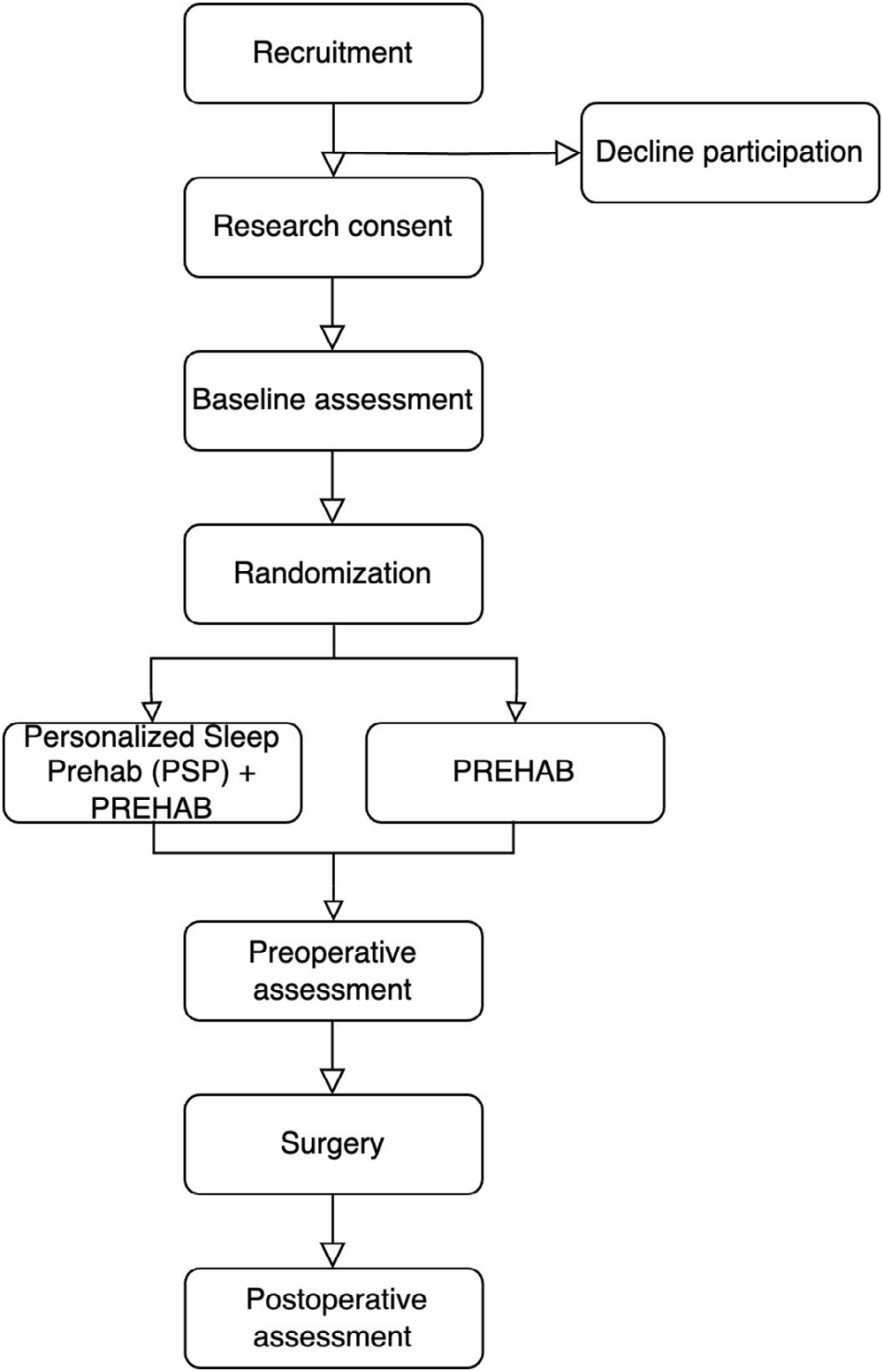
Study flow diagram. PREHAB, multimodal prehabilitation consisting of exercise and nutrition intervention; PSP, personalised sleep prehabilitation. Preoperative assessment occurs within 1 week of surgery. Postoperative assessment occurs 6 weeks after surgery (±1 week).

This is a randomised trial conducted at the surgical Prehabilitation Program at the University Health Network (UHN), a large tertiary academic hospital network in Toronto, ON. Participants will be randomised 1:1 to intervention (PSP+PREHAB): active comparator (PREHAB). Flow of participants is depicted in Figure 1. The initial study protocol has been approved by the University Health Network Research Ethics Board. The trial is registered with ClinicalTrials.gov (ID NCT06762639). This protocol was reported in adherence with the Standard Protocol Items: Recommendations for Interventional Trials (SPIRIT) checklist (SPIRIT 2013 Checklist: Recommended Items to Address in a Clinical Trial Protocol and Related Documents n.d.). The checklist is available in the Supporting Information (Appendix S2).

### 3.2 Participants

Participants will be eligible if they are referred to the UHN Prehabilitation Program; this includes adults (18 years of age or older) referred by a healthcare provider due to elevated surgical risk (most commonly due to frailty). Other inclusion criteria are as follows: (i) surgery date is 4–12 weeks from the time of consent, and (ii) having existing sleep disturbance. An operational definition of sleep disturbance is available in Appendix S3. Exclusion criteria are as follows: (i) an existing diagnosed sleep disorder that is poorly managed or requires referral to a sleep clinician, (ii) currently a shift worker (work schedule outside of 7 am–6 pm), (iii) plans to travel 3 or more hours outside of their usual time zone, (iv) does not have English proficiency, and (v) has a cognitive disability that significantly limits ability to respond to screening questions or adhere to the intervention.

### 3.3 Sample Size

A total of 154 participants will be recruited for this study. The sample size was computed by a biostatistician at UHN for an ANCOVA design in which the Pittsburgh Sleep Quality Index (PSQI) score will be compared between the two arms at the preoperative timepoint, controlling for baseline scores. Assuming a PSQI score mean of 11.9 (SD 2.6) points for the usual care arm (Atoui et al. 2023) with no correlation between baseline and preoperative scores, a sample size of 64 participants per arm (128 total) will be required to detect a difference in mean PSQI score of 1.3 points, which is the minimum clinically important difference (Longo et al. 2021). This difference between the two arms corresponds to a Cohen's d effect size of 0.5 with 80% power and an alpha of 0.05. The final sample accounts for 20% attrition.

### 3.4 Sampling and Recruitment

Participants will be recruited from the Prehabilitation Program at Toronto General Hospital based on the usual referral criteria. Patients who are eligible for the UHN Prehabilitation Program are those with a scheduled surgery at UHN and referred by a member of their circle of care. Upon referral, a member of the patient's circle of care will briefly introduce the study and inquire if the patient is interested in hearing more. If agreeable, a member of the research team will contact the patient to discuss the study protocol, potential risks and benefits to participation and answer patient questions. If the patient is interested, the research team member will initiate the informed consent process.

### 3.5 Randomisation and Blinding

Participants will be randomised 1:1 (PSP+PREHAB: PREHAB) using a computer‐generated randomisation sequence. This randomisation process will be blinded such that the research team member who generates the randomisation sequence is not in contact with interventionists, outcome assessors or the participant's circle of care. Due to the nature of the study, participant and interventionist (PSP) blinding is not possible. However, outcome assessors and clinicians delivering PREHAB will be blinded to group allocation.

### 3.6 Standard of Care Prehabilitation (PREHAB)

All participants (both study arms) will receive standard of care multimodal prehabilitation (PREHAB) from consent until surgery. All participants will undergo a baseline assessment consisting of a verbal interview and physical function assessment to ascertain medical history, functional capacity and relevant information required to individualise PREHAB (Table 1). The PREHAB model of care has been published previously (Randall et al. 2024). PREHAB consists of exercise prehabilitation and nutritional prehabilitation for all participants. Psychological and smoking cessation prehabilitation is delivered to patients screening positive using standardised screening tools. Prehabilitation modalities are summarised below and detailed in Appendix S4.

**TABLE 1:** PREHAB baseline assessment.

Assessment component	Description
Health history and intervention needs screen	Comorbidity collection (CCI), Frailty (Edmonton Frail Scale; EFS), Nutrition risk via the CNST Sleep via the Global Sleep Assessment Questionnaire (GSAQ).
Body composition	Body mass in kilogrammes using a standing digital scale Height in centimetres using a wall‐mounted stadiometer Body mass index (calculated) body fat %, lean mass and fat mass measured with the mBCA 514 (Seca, Hamburg, Germany)
Functional capacity	Grip strength via hand grip dynamometer Timed up and go Short physical performance battery Six‐minute walk test

Exercise Prehabilitation: Exercise‐based prehabilitation will be instructed and delivered by a Registered Kinesiologist. The exercise prescription will be tailored to the results and observations obtained during the baseline assessment. The aerobic training will be light to moderate intensity continuous training (MICT) at 40%–70% of heart rate reserve (HRR, estimated based on age‐predicted HR_max_ and resting heart rate) or approximately four to six on the 10‐point rating of perceived exertion (RPE) scale. The default modality of aerobic exercise is walking. Muscular fitness will be targeted through a minimum of 3–5 resistance training exercises per session targeting major muscle groups (4–6/10 RPE). A standard linear progression will be targeted using the goal of achieving 3 sets of 8–15 repetitions per exercise with a minimum of 24 h of recovery between resistance training sessions. Progression in resistance intensity will occur when 15 repetitions of a given exercise can be completed with only mild exertion (< 4/10 RPE). All exercises will be demonstrated in the Prehabilitation Program facility where participants will have an opportunity to practise and receive feedback or alternate exercises. Exercise prescriptions will target a total volume of 150 min of moderate to vigorous aerobic exercise and 2 sessions of resistance training per week, though this will be individualised based on the results of the baseline assessment. Exercise prescriptions are intended to be completed unsupervised and in the home, but will be modified to support supervised delivery or completion in other settings (e.g., condominium gym).

Nutritional prehabilitation: A registered dietitian (RD) will provide an individualised nutrition assessment and counselling session within the first week of prehabilitation and again in the week prior to surgery (if indicated). Each consultation will be 30–60 min in length and can be conducted in person or via telephone. The patient's nutritional history (via 3‐day diet record), assessment of usual intake and weight history will be reviewed to help identify any nutritional issues or concerns. The session will focus on the goals of nutrition during prehabilitation and in the post‐surgical period by providing strategies to help the patient optimise or enhance the nutritional quality of the diet, maintain a healthy weight and minimise weight gain or weight loss, and address any nutrition‐related questions or concerns. Participants will be informed of the healthy eating recommendations described by the Canada Food Guide. Participants will also be educated on maintaining and/or intaking adequate protein levels (1.2 g/kg/day) to mitigate age‐related muscle depletion and support optimal muscle health. The participant will be encouraged to contact the RD as needed during treatment for ongoing support of any nutrition‐related questions or concerns.

Psychological prehabilitation: If indicated, within 1 week of initiating prehabilitation, a Clinical Psychologist or trainee in supervised practice will deliver a 30–60‐min psychoeducation session that focuses on stress management via relaxation, mindfulness, goal setting and strategies to overcome barriers to practice. In the week prior to surgery, participants will be offered a second consultation with the psychology team member to review their stress management experiences and provide further support for the acute perioperative period. These sessions will also incorporate behaviour change support counselling, based on theoretical models such as Motivational Interviewing, the Trans‐Theoretical Model, Social Cognitive Theory and the Theory of Planned Behaviour (Prochaska and Di Clemente 1982; Bandura 1977; Ajzen 1991). Psychology sessions may be delivered in person or remotely via telephone or Microsoft Teams (Microsoft Corporation, Redmond, WA, USA).

Smoking Cessation: Participants who smoke will be provided with education regarding the impact of preoperative smoking on surgical recovery. Participants will be referred to UHN's smoking cessation programme for one‐on‐one counselling using non‐pharmacological and pharmacological smoking cessation techniques delivered by a pharmacist.

### 3.7 Personalised Sleep Prehabilitation (PSP)

In addition to the standard of care multimodal prehabilitation described above, intervention arm participants will also receive a PSP intervention. The ultimate goal of the PSP is to improve patient sleep health encompassing regularity, quantity and quality (Gunn et al. 2019). The main element of the PSP is BBTI. The 4 intervention sessions will follow the content of BBTI as outlined by Gunn et al. (2019) and Troxel et al. (2012). A participant‐facing researcher‐developed BBTI package will be provided to participants that outlines the crucial components of the intervention (Buysse et al. 2011; Germain et al. 2006). The intervention will be modified such that it can be delivered entirely remotely to ensure feasibility at our site and promote equitable access to health resources. In addition to BBTI, the intervention will consist of sleep hygiene and behaviour change modification. The intervention will be delivered once weekly for 4 weeks lasting 20–60 min per session and delivered one on one. The intervention will be delivered by a regulated health professional (RKin or CPsych) with training and experience delivering primary care sleep intervention. The PSP is tailored to the needs, preferences and context of the participant as identified during the baseline sleep assessment and using a shared decision‐making approach between the research member and participant. These intervention components are described further below.

Baseline assessment: The baseline assessment will consist of a review of participant responses to the Global Sleep Assessment Questionnaire (GSAQ). Results of the screening will be used to initiate a discussion regarding participant sleep habits and behaviour.

BBTI: A research team member will deliver the BBTI intervention. BBTI consists of four main behavioural modifications: reduce time in bed, standardise sleep and wake times, go to bed only when sleepy and stay in bed only if asleep (i.e., stimulus control and sleep restriction) (Gunn et al. 2019). An overview of the four sessions is described in Appendix S5. BBTI was developed to address the shortcomings of CBT‐I whilst building on its success as a first line treatment for insomnia. Delivery of BBTI does not require specialised sleep medicine training. BBTI offers a simple, acceptable and effective treatment that can be implemented in a variety of medical settings. The efficacy of BBTI has been established in older adults and insomnia patients via a synthesis of RCTs (Chen et al. 2023). The BBTI will be delivered remotely or in person as determined through discussion with the participant and research team member.

Sleep hygiene education: Participants will be asked to adhere to 10 sleep hygiene principles that have been shown to improve sleep (e.g., reduce afternoon/evening caffeine intake; Appendix S6). Sleep hygiene consists of adjustments to sleep‐related behaviour and environment. Participants will be provided with a written document outlining each principle.

Behaviour change techniques: The PSP will use behaviour change techniques previously shown to be important for health behaviour change: (Rayward et al. 2020) goal‐setting, social support, self‐monitoring, feedback and graded tasks. The participant in collaboration with the researcher will set sleep‐related SMART (specific, measurable, attainable, realistic and time‐bound) goals. To mobilise the social environment, the participant will be asked to share their goal with one other person. The research team member will also use self‐monitoring, feedback and graded tasks (i.e., behaviour change techniques) to facilitate adherence to the BBTI and sleep hygiene components. For example, participants may start with a focus on five sleep hygiene principles before incorporating the rest. The wrist‐worn activity tracker will also be used for self‐monitoring, feedback and graded tasks. Each behaviour change component will seek to establish behavioural intentions and improve self‐efficacy, the participant's confidence in their ability to change behaviour. The techniques will also be used to minimise attrition.

## 4 Study Outcomes

Participant data will be collected at the baseline assessment, within 1 week prior to surgery and at 6 weeks postoperatively. Programme measures are classified as (1) screening, (2) sample characteristics, (3) sleep outcomes, (4) clinical outcomes, and (5) physical fitness, anthropometric and patient‐reported measures (Appendix S7). A timeline of sleep health data collection and time points is shown in Table 2. This study is designed to make efficient use of participant time and minimise appointment burden by using standard prehabilitation assessment time points.

**TABLE 2:** Timeline of sleep outcomes.

		T0	T1	—	T2
	Screening	Baseline	1 week preop	Surgery	6 week postop
Recruitment	×				
Attrition		×	×		×
Safety		×	×		×
Screening					
Global Sleep Assessment Questionnaire (GSAQ)	×				
Insomnia Severity Index	×				
STOP‐Bang Questionnaire	×				
Restless Legs Syndrome Diagnostic Index	×				
Sleep outcomes					
PSQI (primary outcome)		×	×		×
Sleep diary		×	×		×
PSP adherence*		×	×		
Sleep self‐efficacy scale		×	×		×

*PSP group only.

Eligibility rate will be measured as the number of patients eligible for the study divided by the number of patients screened for eligibility. Recruitment rate will be measured as the proportion of participants who are randomised divided by the number of identified eligible patients. Reasons for non‐participation will be collected. Reasons for, all or part of, the intervention not being delivered as intended will be recorded. The frequency of drop‐out during study participation will be documented including reasons. Any safety or adverse events related to the prehabilitation intervention will be reported during weekly telephone calls with participants. Reporting and grading will follow the Common Terminology Criteria for Adverse Events version 5.0 (U.S. Department of Health and Human Services 2017) Reporting of adherence to the PSP will be calculated as the number of sessions attended divided by the number of sessions stipulated in the protocol. In addition, the research member will record the number of BBTI components the participant adhered to as determined through discussion.

Demographic, physical fitness and anthropometric, clinical and patient‐reported outcomes are described in Appendix S7. Briefly, demographic characteristics will be documented via a standardised questionnaire. Physical fitness and anthropometric outcomes include the Six‐minute Walk Test (6MWT), short physical performance battery, timed up and go, grip strength, body mass index (BMI) and body composition via bioelectrical impedance analysis (mBCA 514, Seca, Hamburg, Germany). Clinical outcomes include hospital length of stay, readmission and surgical complications documented via patient medical record up to 90 days after surgery. Patient‐reported outcomes include the Edmonton Frail Scale (Rolfson et al. 2006) and Canadian Nutrition Screening Tool (CNST) (Laporte et al. 2015) administered at baseline and the PROMIS‐29+2 profile (Fries et al. 2009) administered at all timepoints.

Sleep health outcomes include the PSQI (Buysse et al. 1989) will be used to assess sleep health and is the primary outcome of this trial. The PSQI is a self‐report measure of sleep quality amongst seven subscales including subjective sleep quality, sleep latency, sleep duration, habitual sleep efficiency, sleep disturbances, sleep medication and daytime dysfunction. The scale's validity and reliability have been determined amongst clinical and older adult populations (Spira et al. 2012; Carpenter and Andrykowski 1998). The GSAQ (Roth et al. 2002) will be used to screen for sleep disorders. A previous systematic review (Klingman et al. 2017) identified the GSAQ as the most suitable screening tool for multiple sleep disorders due to its efficiency and comprehensiveness. The Insomnia Severity Index (ISI) will be used to screen for insomnia, the most common sleep disorder. The validity and reliability of the ISI have been previously established (Bastien et al. 2001). The STOP‐Bang Questionnaire will be used to screen for obstructive sleep apnea. The validity and reliability of this instrument have been previously established in surgical patients (Chung et al. 2016). The Restless Legs Syndrome Diagnostic Index (RLS‐DI) will be used to screen for Restless Legs Syndrome (Beneš and Kohnen 2009). This scale has been previously validated with high sensitivity and specificity to screen for Restless Legs Syndrome (cut‐off score > 11). The sleep self‐efficacy scale (SES) measures confidence in sleep behaviour change (Lacks 1987). Self‐efficacy has previously been determined as one of the most significant predictors of adherence to a behavioural intervention for improving sleep (Bluestein et al. 2011). A participant sleep diary will be used to record sleep for 1 week at each study time point (Carney et al. 2012). The sleep diary will be available in hard copy, or a virtual copy may be emailed to the participant if requested. The questions will be based on a subset of questions available online at https://consensussleepdiary.com/.

All participants will be provided with a Fitbit Inspire 3, a small non‐intrusive and waterproof wrist‐worn device that uses actigraphy to measure orientation and movement and uses photoplethysmography (PPG) to detect periodic changes in blood flow beneath the sensor; thereby measuring changes in heart rate. The activity tracker measures sleep–wake activity to determine time in bed, sleep duration, nighttime awakenings and physical activity levels. The heart rate monitor will measure resting heart rate and heart rate variability, metrics of overall fitness and autonomic nervous system function that have previously been found to be correlated with sleep (Sajjadieh et al. 2020). The device's predecessor was validated against polysomnography demonstrating high sensitivity (93.9%) and low specificity (13.1%), with an accuracy of 76% (Lim et al. 2023).

### 4.1 Analytic Plan

All data will be collected and imported into R version 4.1.2 for statistical analysis; an alpha of 0.05 will be used. Participant characteristics will be summarised using means (SD) or medians [IQR] for continuous variables and frequency (%) for categorical variables. We will report reasons for exclusion and attrition, as well as safety or adverse events with frequency and percentages.

The statistical analysis plan was created in consultation with a UHN biostatistician. The primary outcome (PSQI score) will be compared between the two treatment arms using analysis of covariance (ANCOVA), controlling for baseline PSQI score. A linear mixed effects model will be used to compare treatment arms with respect to the longitudinal PSQI scores (baseline, preoperative, 6 weeks post‐surgery). Point estimates and 95% confidence intervals will be calculated for changes in the PSQI between groups at each timepoint. We will use Q–Q plots and histograms to assess data normality. If non‐normality is encountered and cannot be corrected through data transformations (e.g., log, square root), we will employ appropriate non‐parametric alternatives, such as the Mann–Whitney U test for between‐group comparison. Model assumptions including homogeneity of variance (Levene's Test) and normality of dependent variables (Shapiro–Wilk Test) will be used. Independence of observations was assessed using the Durbin Watson (DW) statistic, with values between 1.5 and 2.5 indicating independence. Sensitivity analyses will address potential loss to follow‐up by replacing the missing outcome measurements with best or worst values to assess the robustness of our results. In the unlikely event of unbalanced baseline characteristics between the treatment arms, the mixed effects models will be adjusted for these potential confounders (e.g., age, sex, etc.). Conclusions will be drawn using comparisons of each time point between groups. The minimum clinically important difference for the PSQI score will be 1.3 based on previous research (Shergis et al. 2016). Given a lack of consensus for the PSQI MCID, previous research has interpreted any statistically significant change in the PSQI to be clinically meaningful (Shergis et al. 2016).

Additional exploratory analyses using linear mixed effect models will be conducted to understand the effect of the intervention on secondary outcomes that are measured longitudinally, including physical fitness (e.g., 6MWT) outcomes and the PROMIS‐29+2. For outcomes that are measured at only one time point (e.g., length of hospital stay), we will use non‐parametric Wilcoxon signed‐rank *t*‐tests or Chi‐square tests for continuous and categorical outcomes, respectively. Given the exploratory nature of secondary outcomes, *p*‐values will be adjusted using Bonferroni corrections to account for multiple comparisons. All analyses will be conducted using the intention to treat principle. The data will be reported in accordance with the Consolidated Standards of Reporting Trials (CONSORT) guidelines for reporting randomised trials (Schulz et al. 2010).

## 5 Discussion

As effectiveness, implementation and economic data supporting prehabilitation grows, continued advancements are warranted (Randall et al. 2024). This protocol describes a randomised trial aiming to expand the delivery of prehabilitation to include a novel intervention modality. Findings will inform the integration of sleep assessment, measurement and intervention in the preoperative setting.

The relationship between sleep health and overall health is well documented, and several studies have demonstrated the importance of sleep for surgical recovery. Capitalising on the preoperative window to address surgical risk factors has significantly improved patient care in recent decades (McIsaac et al. 2022). Within our clinically integrated prehabilitation service in Toronto, Canada, we have recognised the significant prevalence of sleep disruption as a limitation to functional capacity.

Although at least 47 interventions have been initiated preoperatively and measured a sleep outcome, (Sibley et al. 2024) to our knowledge, no study has evaluated the effectiveness of a sleep‐specific intervention preoperatively. Very few studies within the prehabilitation literature have acknowledged the importance of sleep. In 2023, Atoui and colleagues (2023) conducted a pilot RCT evaluating the effect of home‐based multimodal prehabilitation on sleep health versus usual care (Atoui et al. 2023). The intervention did not demonstrate improvements to within‐ or between‐group self‐reported (PSQI) or device‐measured sleep at any time point. As such, despite the well‐documented relationships between exercise, nutrition, and psychological stress (i.e., standard multimodal prehabilitation), these strategies may not sufficiently address disturbed preoperative sleep health (Kekecs et al. 2014; Knoerl et al. 2022). The PSP intervention was curated to be integrated into the prehabilitation workflow at our institution. The protocol also builds on other studies that have successfully integrated digital technology and behaviour change techniques into prehabilitation interventions (Blumenau Pedersen et al. 2023; Fong et al. 2023). The BBTI is the main component of the intervention. Not only is this an evidence‐based intervention with proven efficacy, but it was designed to be delivered by non‐experts (Gunn et al. 2019). As such, this intervention is generalisable to other prehabilitation settings.

Some strengths of this study include the randomised design with outcome assessors blinded to group allocation. The study will integrate and expand on a gold‐standard multimodal prehabilitation intervention including exercise, nutrition, psychological intervention and smoking cessation delivered by regulated health professionals with several years of experience delivering prehabilitation. The study will capture robust outcomes including demographic, physical and anthropometric, sleep health, clinical and patient‐reported outcomes. The analysis will be conducted using intention to treat. There are limitations to this study worthy of discussion. Selection bias will favour the surgical subgroups referred to the Prehabilitation Program during study recruitment as well as the physicians and patients who refer and accept referral to prehabilitation, respectively. The study will lack a true control group without intervention. This is warranted given the overwhelming evidence supporting prehabilitation. Given that the intervention is delivered by an interprofessional team of allied health professionals, the generalisability of this study to other lower‐resource settings can be considered a limitation. Finally, this study is being conducted without any formal published feasibility trials. As such, it is difficult to predict participant adherence to or satisfaction with the intervention. However, the sleep measures included within this protocol are being used in an ongoing prospective observational study of the same target population with acceptable rates of recruitment, satisfaction and data completeness.

## 6 Conclusion

Prehabilitation is a health intervention with growing support for improving surgical care. The proposed study will be the first to explore the effect of a PSP in addition to standard‐of‐care prehabilitation service on sleep health and surgical recovery. Results will determine if the effectiveness of prehabilitation can be improved with an individualised sleep prehabilitation intervention. As such, the present study will contribute important data regarding the modifiability of preoperative sleep health through targeted and individualised intervention.

## Author Contributions

**Daniel Sibley:** conceptualization, funding acquisition, writing – original draft, methodology. **Ian Randall:** funding acquisition, methodology, writing – review and editing, supervision. **S. Nicole Culos‐Reed:** funding acquisition, methodology, writing – review and editing, supervision. **P. Maxwell Slepian:** funding acquisition, writing – review and editing. **Mandeep Singh:** funding acquisition, methodology, writing – review and editing, supervision. **Daniel Santa Mina:** funding acquisition, methodology, writing – review and editing, supervision.

## Ethics Statement

This study protocol was reviewed and approved by the University Health Network Research Ethics Board. Protocol number (version date): 24‐5450 (December 17, 2024).

## Consent

Written informed consent will be obtained for all study participants.

## Conflicts of Interest

Daniel Santa Mina is a Partner/Founder of Prehab Consultants Inc.

## Supporting information


**Data S1:** Supporting Information.

## Data Availability

The data that support the findings of this study are available from the corresponding author upon reasonable request.
