# Supplementary material for: Evaluating the Effect of a Sleep Prehabilitation Intervention in Patients Awaiting Elective Surgery: Protocol for a Single‐Blind Randomised Trial
Source: J Sleep Res. 2025 Aug 30;35(2):e70173. doi: 10.1111/jsr.70173 (PMC13003284; doi:10.1111/jsr.70173)
Supplement: Supplementary file 1 — Data S1: Supporting Information. [file JSR-35-e70173-s001.pdf]

Supplemental Material

Appendix S1: Research questions
Appendix S2: SPIRIT 2013 Checklist
Appendix S3: Sleep disturbance definition
Appendix S4: Standard of care multimodal prehabilitation description (PREHAB)
Appendix S5: BBTI Intervention
Appendix S6: Sleep hygiene education
Appendix S7: Secondary Outcomes Description and Timeline of Measurement

Appendix S1: Research questions

Table S1: Study research questions

Primary Research Question	Does participation in a personalized sleep prehabilitation intervention in addition to standard-of-care surgical prehabilitation (PSP+PREHAB) improve preoperative, self-reported sleep health compared to standard-of-care surgical prehabilitation (PREHAB)?
Secondary Research Questions	Does PSP+PREHAB improve postoperative self-reported sleep health compared to PREHAB?
	Does participation in PSP+PREHAB improve anthropometric, physical fitness, patient-reported, or clinical outcomes compared to PREHAB?
	Does participation in PSP+PREHAB or PREHAB improve sleep health (preoperatively or postoperatively) relative to baseline?
	Does participation in a PSP intervention in addition to standard-of-care surgical prehabilitation (PSP+PREHAB) or standard-of-care surgical prehabilitation (PREHAB) improve anthropometric, physical fitness, patient-reported, or clinical outcomes preoperatively relative to baseline?
	What is the prevalence of disturbed sleep among patients referred to UHN's surgical prehabilitation program?
	Is participant sleep at baseline related to surgical outcomes following participation in surgical prehabilitation in the PREHAB or PSP+PREHAB group?
	Is change in participant sleep preoperatively related to surgical outcomes?

Appendix S2: SPIRIT 2013 Checklist

SPIRIT — Standard Protocol Items: Recommendations for Interventional Trials

SPIRIT 2013 Checklist: Recommended items to address in a clinical trial protocol and related documents*

Section/item	Item No	Description	Addressed on page number
**Administrative information**			
Title	1	Descriptive title identifying the study design, population, interventions, and, if applicable, trial acronym	Page 1 (Title page)
Trial registration	2a	Trial identifier and registry name. If not yet registered, name of intended registry	Page 1 (Title page)
	2b	All items from the World Health Organization Trial Registration Data Set	Trial registration
Protocol version	3	Date and version identifier	Page 1 (Title page)
Funding	4	Sources and types of financial, material, and other support	Page 1 (Title page)
Roles and responsibilities	5a	Names, affiliations, and roles of protocol contributors	Page 1 (Title page)
	5b	Name and contact information for the trial sponsor	Page 1 (Title page)
	5c	Role of study sponsor and funders, if any, in study design; collection, management, analysis, and interpretation of data; writing of the report; and the decision to submit the report for publication, including whether they will have ultimate authority over any of these activities	Page 13
	5d	Composition, roles, and responsibilities of the coordinating centre, steering committee, endpoint adjudication committee, data management team, and other individuals or groups overseeing the trial, if applicable (see Item 21a for data monitoring committee)	NA
**Introduction**			

Background and rationale	6a	Description of research question and justification for undertaking the trial, including summary of relevant studies (published and unpublished) examining benefits and harms for each intervention	Page 3-4 (Introduction)
	6b	Explanation for choice of comparators	Page 3-4 (Introduction)
Objectives	7	Specific objectives or hypotheses	Page 3-4 (Introduction); Appendix A
Trial design	8	Description of trial design including type of trial (eg, parallel group, crossover, factorial, single group), allocation ratio, and framework (eg, superiority, equivalence, noninferiority, exploratory)	Page 4 (Study design and setting)
**Methods: Participants, interventions, and outcomes**			
Study setting	9	Description of study settings (eg, community clinic, academic hospital) and list of countries where data will be collected. Reference to where list of study sites can be obtained	Page 4 (Study design and setting)
Eligibility criteria	10	Inclusion and exclusion criteria for participants. If applicable, eligibility criteria for study centres and individuals who will perform the interventions (eg, surgeons, psychotherapists)	Page 5-6 (Participants)
Interventions	11a	Interventions for each group with sufficient detail to allow replication, including how and when they will be administered	Page 5-8 (PRHEAB AND PSP)
	11b	Criteria for discontinuing or modifying allocated interventions for a given trial participant (eg, drug dose change in response to harms, participant request, or improving/worsening disease)	Page 5-8 (PRHEAB AND PSP)
	11c	Strategies to improve adherence to intervention protocols, and any procedures for monitoring adherence (eg, drug tablet return, laboratory tests)	Page 5-8 (PRHEAB AND PSP)
	11d	Relevant concomitant care and interventions that are permitted or prohibited during the trial	Page 5-8 (PRHEAB AND PSP)

Outcomes	12	Primary, secondary, and other outcomes, including the specific measurement variable (eg, systolic blood pressure), analysis metric (eg, change from baseline, final value, time to event), method of aggregation (eg, median, proportion), and time point for each outcome. Explanation of the clinical relevance of chosen efficacy and harm outcomes is strongly recommended	Page 8-9 (Study outcomes)
Participant timeline	13	Time schedule of enrolment, interventions (including any run-ins and washouts), assessments, and visits for participants. A schematic diagram is highly recommended (see Figure)	Page 8-9 (Study outcomes); Appendix F
Sample size	14	Estimated number of participants needed to achieve study objectives and how it was determined, including clinical and statistical assumptions supporting any sample size calculations	Page 5 (Sample size)
Recruitment	15	Strategies for achieving adequate participant enrolment to reach target sample size	Page 5 (Sampling and recruitment)
**Methods: Assignment of interventions (for controlled trials)**			
Allocation:			
Sequence generation	16a	Method of generating the allocation sequence (eg, computer-generated random numbers), and list of any factors for stratification. To reduce predictability of a random sequence, details of any planned restriction (eg, blocking) should be provided in a separate document that is unavailable to those who enrol participants or assign interventions	Page 5 (Randomizati on and Blinding)
Allocation concealment mechanism	16b	Mechanism of implementing the allocation sequence (eg, central telephone; sequentially numbered, opaque, sealed envelopes), describing any steps to conceal the sequence until interventions are assigned	Page 5 (Randomizati on and Blinding)
Implementation	16c	Who will generate the allocation sequence, who will enrol participants, and who will assign participants to interventions	Page 5 (Randomizati on and Blinding)
Blinding (masking)	17a	Who will be blinded after assignment to interventions (eg, trial participants, care providers, outcome assessors, data analysts), and how	Page 5 (Randomizati on and Blinding)

	17b	If blinded, circumstances under which unblinding is permissible, and procedure for revealing a participant's allocated intervention during the trial	Page 5 (Randomization and Blinding)

Methods: Data collection, management, and analysis

Data collection methods	18a	Plans for assessment and collection of outcome, baseline, and other trial data, including any related processes to promote data quality (eg, duplicate measurements, training of assessors) and a description of study instruments (eg, questionnaires, laboratory tests) along with their reliability and validity, if known. Reference to where data collection forms can be found, if not in the protocol	Page 8-9 (Study outcomes); Study protocol
	18b	Plans to promote participant retention and complete follow-up, including list of any outcome data to be collected for participants who discontinue or deviate from intervention protocols	Page 8-9 (Study outcomes)
Data management	19	Plans for data entry, coding, security, and storage, including any related processes to promote data quality (eg, double data entry; range checks for data values). Reference to where details of data management procedures can be found, if not in the protocol	Study protocol
Statistical methods	20a	Statistical methods for analysing primary and secondary outcomes. Reference to where other details of the statistical analysis plan can be found, if not in the protocol	Page 9-10 (Analytic plan)
	20b	Methods for any additional analyses (eg, subgroup and adjusted analyses)	Page 9-10 (Analytic plan)
	20c	Definition of analysis population relating to protocol non-adherence (eg, as randomised analysis), and any statistical methods to handle missing data (eg, multiple imputation)	Page 9-10 (Analytic plan)

Methods: Monitoring

Data monitoring	21a	Composition of data monitoring committee (DMC); summary of its role and reporting structure; statement of whether it is independent from the sponsor and competing interests; and reference to where further details about its charter can be found, if not in the protocol. Alternatively, an explanation of why a DMC is not needed	Study protocol

	21b	Description of any interim analyses and stopping guidelines, including who will have access to these interim results and make the final decision to terminate the trial	Study protocol
Harms	22	Plans for collecting, assessing, reporting, and managing solicited and spontaneously reported adverse events and other unintended effects of trial interventions or trial conduct	Page 9-10 (Analytic plan)
Auditing	23	Frequency and procedures for auditing trial conduct, if any, and whether the process will be independent from investigators and the sponsor	Study protocol
**Ethics and dissemination**			
Research ethics approval	24	Plans for seeking research ethics committee/institutional review board (REC/IRB) approval	Study protocol
Protocol amendments	25	Plans for communicating important protocol modifications (eg, changes to eligibility criteria, outcomes, analyses) to relevant parties (eg, investigators, REC/IRBs, trial participants, trial registries, journals, regulators)	Study protocol
Consent or assent	26a	Who will obtain informed consent or assent from potential trial participants or authorised surrogates, and how (see Item 32)	Study protocol
	26b	Additional consent provisions for collection and use of participant data and biological specimens in ancillary studies, if applicable	NA
Confidentiality	27	How personal information about potential and enrolled participants will be collected, shared, and maintained in order to protect confidentiality before, during, and after the trial	Study protocol
Declaration of interests	28	Financial and other competing interests for principal investigators for the overall trial and each study site	Page 14
Access to data	29	Statement of who will have access to the final trial dataset, and disclosure of contractual agreements that limit such access for investigators	Page 14
Ancillary and post-trial care	30	Provisions, if any, for ancillary and post-trial care, and for compensation to those who suffer harm from trial participation	Page 14

Dissemination policy	31a	Plans for investigators and sponsor to communicate trial results to participants, healthcare professionals, the public, and other relevant groups (eg, via publication, reporting in results databases, or other data sharing arrangements), including any publication restrictions	Study protocol
	31b	Authorship eligibility guidelines and any intended use of professional writers	Page 14
	31c	Plans, if any, for granting public access to the full protocol, participant-level dataset, and statistical code	Page 14
**Appendices**			
Informed consent materials	32	Model consent form and other related documentation given to participants and authorised surrogates	Study protocol
Biological specimens	33	Plans for collection, laboratory evaluation, and storage of biological specimens for genetic or molecular analysis in the current trial and for future use in ancillary studies, if applicable	NA

*It is strongly recommended that this checklist be read in conjunction with the SPIRIT 2013 Explanation & Elaboration for important clarification on the items. Amendments to the protocol should be tracked and dated. The SPIRIT checklist is copyrighted by the SPIRIT Group under the Creative Commons "Attribution-NonCommercial-NoDerivs 3.0 Unported" license.

Appendix S3: Sleep disturbance definition

Sleep disturbance is operationally defined as a patient self-reporting one of the following:

- < 7 hours of sleep on most nights in the past month
- a sleep latency > 30 minutes on most nights in the past month
- wake up throughout the night on most nights in the past month
- wake up earlier than intended on most nights in the past month
- experience daytime sleepiness or fatigue on most days in the past month
- dissatisfaction with sleep quality on most nights in the past month

Appendix S4: Standard of care multimodal prehabilitation description (PREHAB)

All participants will undergo a baseline assessment to obtain the information necessary for prehabilitation. The assessment consists of health history and intervention needs screen, functional capacity, and body composition. The functional capacity assessment will consist of a grip strength test via hand grip dynamometer, Timed Up and Go, and the 6-Minute Walk Test (6MWT).

Exercise Prehabilitation: Exercise-based prehabilitation will be instructed and delivered by a Registered Kinesiologist. This will include individualized, **light- to moderate-**intensity resistance training and aerobic exercise components. The exercise prescription will be tailored to the results and observations obtained during the baseline assessment. All exercises will be instructed and demonstrated in the prehabilitation program facility where participants will have an opportunity to practice and receive feedback/corrections or alternate exercises. The selected aerobic exercise modalities will be made, in part, by the participant's ability, potential risk for injury, and available home-based equipment. Each exercise prescription will target a total volume of 150 minutes of moderate to vigorous aerobic exercise and 2 sessions of resistance training per week. The distribution of aerobic and resistance exercises will be individually tailored such that participants may complete durations of exercise that are tolerable without over-exerting (i.e., 30 minutes of daily exercise may be divided into 2-3 shorter duration bouts). While exercise prescriptions are intended to be completed unsupervised and in the home; exercise programming in settings local/convenient to the participant will be supported with programming adaptations (e.g., access to a condominium gym or community fitness facility). Each prescribed session will include: a minute warm-up, aerobic exercise, resistance training, and a cool-down, but may be modified to accommodate the participant's exercise ability (e.g., separate aerobic and resistance training sessions, brief bouts of training occurring two or times per day).

The aerobic training will be conducted via light to moderate intensity continuous training (MICT) at 40-70% of heart rate reserve (HRR, estimated based on age-predicted HR_{max} and resting heart rate) or approximately four to six on the 10-point rating of perceived exertion scale (RPE).(Borg, 1985; Noble et al., 1983) The default modality of training is walking; and we have also previously used a low-intensity aerobic step class video for older men awaiting prostate cancer surgery (developed by our team and previously used by our team in prehabilitation research: https://www.youtube.com/watch?v=sMsDsuC98gc&list=PLuue1U2fPzkCV5LabKQ17d1HCQvbkTWFU). Alternatively, participants may utilize other available equipment or facilities that can elicit the prescribed aerobic training dose (e.g., stationary cycling, stepping, rowing, swimming, etc.). Participants will be educated on how to rate their exertion via manual heart rate testing and rating of perceived exertion (with additional instruction in the provided manual). General musculoskeletal fitness will be targeted through a minimum of 3-5 resistance training exercises per session targeting the major muscle groups (e.g., shoulders, chest, upper/lower back, core, upper/lower legs). A standard linear progression will be targeted using the goal of achieving 3 sets of 8-12 repetitions per exercise with a minimum of 24 hours of recovery between resistance training sessions. Progression in resistance intensity will occur when 15 repetitions of a given exercise can be completed with only mild exertion. Participants will be provided with resistance bands and a stability ball to perform resistance training at home if needed. Exercise staff will communicate with prehabilitation participants via telephone and/or email with the participants weekly to ensure program compliance, record adherence, support appropriate progression, and to address to any barriers to exercise that may prevent participation.

Nutritional prehabilitation: A registered dietitian (RD) will provide an individualized nutrition assessment and counselling session within the first week of prehabilitation and again in the week prior to surgery. Each consultation will be ~ 60 minutes in length and can be conducted in person or via telephone. The patient's nutritional history (via 3-day diet record), assessment of usual intake and weight history will be reviewed to help identify any nutritional issues/concerns. The session will focus on the goals of nutrition during prehabilitation and in the post-surgical period by providing strategies to help the patient optimize or enhance the nutritional quality of the diet, maintain a healthy weight and minimize weight gain or weight loss, and address any nutrition-related questions or concerns. Participants will be informed of the healthy eating recommendations described by the Canada Food guide(Health Canada, 2019) The updated 2019 guidance document promotes healthy eating and overall nutritional well-being with an emphasis on choosing and identifying nutritious foods; highlighting important food skills to support healthy eating; and creating a supportive environment for healthy eating. Participants will also be educated on maintaining and/or intaking adequate protein levels. For older adults, it is recommended at least 1.2 g/kg/day to mitigate age-related muscle depletion and support optimal muscle health.(Phillips et al., 2016) In the surgical setting, patients with low reserve, including malnourished, frail, and sarcopenia are vulnerable with diminished capacity to respond to the added demands of the surgical insult.(Amrock & Deiner, 2014; Malietzis et al., 2016) To help maintain dietary protein sufficiency for exercise and prevent catabolism associated with surgery, participants with inadequate protein intake will be recommended to consume 26g of whey protein isolate supplementation (ISOlution, Enhanced Medical Nutrition, Hamilton, CA). Participants will be recommended to consume the supplementation in a beverage or food daily, and ideally within 24 hours of an exercise session as advised by previous nutrition-prehabilitation protocols(Arends et al., 2006; Gillis et al., 2016).. The parrticipant will be encouraged to contact the RD as needed during treatment for ongoing support of any nutrition-related questions or concerns.

Psychological preparation for surgery: Stress management training before surgery has been shown to be effective at reducing depression and fatigue postoperatively.(Garssen et al., 2013) Furthermore, research suggests that psychological distress can be mitigated through brief stress management techniques such as relaxation breathing techniques, guided imagery, meditation techniques, and psychoeducational interventions.(Rao et al., 2009; Tsimopoulou et al., 2015) If indicated, within one week of initiating prehabilitation, a Clinical Psychologist or trainee in supervised practice will deliver a ~60-minute psychoeducation session that focuses on stress management via relaxation, mindfulness, goal setting, and strategies to overcoming barriers to practice. In the week prior to surgery, participants will be offered a second consultation with the psychology team member to review their stress management experiences and provide further support for the acute perioperative period. These sessions will also incorporate behaviour change support counseling, based on theoretical models such as the Motivational Interviewing, Trans-Theoretical Model, Social Cognitive Theory and the Theory of Planned Behaviour. Aligned with these principles, all participants will receive a manual grounded in the above-mentioned behaviour theories successfully used in our previous research. To help participants with daily stress management practice, publicly available links to written and audio-based materials describing mindfulness, progressive muscle relaxation, deep breathing, and visualization will be provided within the manual. Psychology sessions may be delivered in person or via telephone.

Smoking Cessation: Participants who smoke will be provided with education regarding the impact of preoperative smoking on surgical recovery. Participants will be referred to UHN's smoking

cessation program for one-on-one counselling using non-pharmacological and pharmacological smoking cessation techniques delivered by a pharmacist.

More information about prehabilitation can be found at www.uhn.ca/prehab.

Appendix S5: BBTI Intervention

Session	Description
1[a]	• Develop rapport with the participant<br>• Educate participant on mechanisms of sleep (i.e., homeostatic drive and circadian rhythm) and healthy sleep practices (i.e., sleep hygiene)<br>• Discuss the four BBTI[b] sleep components and form a "sleep prescription" (e.g., tailored sleep schedule)<br>• Discuss potential consequences of sleep prescription (e.g., temporary increase in daytime sleepiness)<br>• Motivational interviewing to discuss barriers to adhering to the 4 BBTI sleep components
2	• Review adherence to recommendations and adjust if needed to promote adherence (e.g., time in bed may be increased or decreased based on adherence to recommendations and reported sleep latency and wake after sleep onset)<br>• Provide positive reinforcement for good adherence and support for poor adherence
3	• Continue to review adherence and make adjustments<br>• Provide positive reinforcement for good adherence and support for poor adherence
4	• Review progress, adjust recommendations (e.g., time in bed, sleep scheduling) as needed.<br>• Discuss importance of ongoing monitoring and strategies for relapse prevention<br>• Discuss potential challenges to adhering as surgery approaches

[a] Prior to the first appointment, the interventionist will review participant responses to the sleep questionnaires and sleep diary. The interventionist will also review the participant's electronic medical record.
[b] 1) Reduce time in bed to usual sleep duration + 30 mins. Time in bed is not reduced below 6 hours; 2) Consistent sleep scheduling (emphasis on consistent wake time); 3) Go to be when sleepy; 4) If unable to sleep within 30 minutes, leave the bed to perform a relaxing activity (e.g., reading).
Notes: Intervention adapted from:

Gunn HE, Tutek J, Buysse DJ. Brief Behavioral Treatment of Insomnia. Sleep Med Clin. 2019 Jun;14(2):235-243. doi: 10.1016/j.jsmc.2019.02.003. Epub 2019 Apr 1. PMID: 31029189.
Troxel WM, Germain A, Buysse DJ. Clinical management of insomnia with brief behavioral treatment (BBTI). Behav Sleep Med. 2012 Oct;10(4):266-79. doi: 10.1080/15402002.2011.607200. PMID: 22946736; PMCID: PMC3622949.

Appendix S6: Sleep hygiene education

Sleep Hygiene

Here are a few tips to help you get a better night's sleep

- Adults aged 18-64 are recommended to get 7-9 hours of sleep each night.
- Adults aged 65+ are recommended to get 7-8 hours of sleep each night.

1. Go to sleep and wake up at the same time every day.
2. Limit daytime naps to 20 minutes or less.
3. Make sure your bedroom is completely dark.
4. Try to use your bedroom only for sleep.
5. If you can't sleep, leave your bedroom and perform a relaxing activity.
6. Avoid caffeine in the afternoon and evening.
7. Keep your bedroom cool (18.3°C).
8. Avoid alcohol, cigarettes, and narcotics.
9. Avoid large meals and exercise right before sleep.
10. Add white noise to your bedroom.

UHN Toronto General Toronto Western Princess Margaret Toronto Rehab Michener Institute
COURAGE LIVES HERE

Version date: September 9, 2024

Prevent. Prepare. Prehabilitation

Appendix S7: Secondary Outcomes Description and Timeline of Measurement

Table S2: Description of secondary outcomes

**Standard of Care Measure**	**Tool/Description**
Participant Characteristics	
Frailty	Edmonton Frail Scale (EFS)(Rolfson et al., 2006)
Demographics	Assessed using a standardized questionnaire assessing ethnicity, marital status, education, work status, smoking status, and alcohol consumption
Comorbidities	Charlson Comorbidity Index (CCI).)(Charlson et al., 1994) The CCI contains 19 categories of comorbid conditions and assigns a weighted value to each comorbidity based on risk of death.
Canadian Nutrition Screening Tool (CNST)	Used to determine nutritional status. This tool includes two key items: weight loss and food intake. Responders that indicate both weight loss without trying to lose weight and have been eating less than usual for more than a week suggests nutritional risk. The CNST is a validated and reliable tool that shows good sensitivity and specificity in Canadian hospitals.(Laporte et al., 2015) In addition, a 3-day food record will be used to quantify nutritional intake to aid dietary assessment and recommendations from the dietitian.
Clinical Outcomes	
Postoperative length of hospital stay	Will be recorded from the participant's medical record in days from the time of surgery until hospital discharge.
Discharge destination (i.e., rehabilitation) and total inpatient stay	Will recorded in days from patient's medical record.
Complications including mortality	Will follow the Clavien-Dindo classification.(Clavien et al., 2009) Any health event that requires readmission will also be documented. This will be recorded during hospitalization and within 30 days postoperatively.
Physical fitness, anthropometric, and patient-reported measures	
Grip strength	Via hand grip dynamometer
Timed up and go	Via published protocol(Department of Health et al., n.d.)
Six-minute walk test	Via published protocol(Crapo et al., 2012)
Body composition	Body mass in kilograms using a standing digital scale. Height in centimetres using a wall-mounted stadiometer. Body mass index (calculated) body fat %, lean mass, and fat mass measured with the mBCA 514 (Seca, Hamburg, Germany)

Physical and emotional symptom severity	Will be measured using Patient-Reported Outcomes Measurement Information System (PROMIS-29+2) assessing depression, anxiety, fatigue, physical function, pain interference, sleep disturbance, social participation and cognitive function, 4-6 items for each domain. (Fries et al., 2009; Papuga et al., 2018; Segawa et al., 2020)

Table S3. Timeline of Secondary Outcomes

		T0	T1	--	T2
	**Screening**	**Baseline**	**1 week preop**	**Surgery**	**6 weeks postop**
Patient characteristics and screening					
Frailty Screen (EFS)	×				
Demographics		×			
Comorbidity (CCI)		×			
Nutrition Screen (CNST)		×			
Clinical outcomes					
Clavien-Dindo Surgical Complication (grade)				×	×
Postoperative hospital LOS				×	
Discharge destination				×	
Readmission and ER visits				×	×
Anthropometric, physical fitness and patient reported outcomes					
Height		×	×		×
Weight		×	×		×
Waist circumference		×	×		×
Body fat percent		×	×		×
Body mass index		×	×		×
6-minute walk test		×	×		×
Grip strength		×	×		×
Timed up and go		×	×		×
Short physical performance battery		×	×		×
PROMIS-29+2		×	×		×
Activity tracker					
Physical activity data[a]		×	×		×
Resting heart rate		×	×		×
Heart rate variability		×	×		×

Abbreviations: CCI, Charlson Comorbidity Index; CNST, Canadian Nutrition Screening Tool; DASI; Duke Activity Status Index, EFS, Edmonton Frailty Scale; ER, emergency room; LOS, length of stay; PHQ-9, Patient Health Questionnaire; PROMIS, Patient-Reported Outcomes Measurement Information System. [a]Physical activity data will consist of time (minutes) spent in each intensity: sedentary, light, and moderate-vigorous.
